# Commentary: Meta-Analysis of 3D Printing Applications in Traumatic Fractures

**DOI:** 10.3389/fsurg.2021.783743

**Published:** 2021-11-11

**Authors:** Som P. Singh, Kevin J. Varghese, Fahad M. Qureshi

**Affiliations:** Department of Biomedical Sciences, University of Missouri—Kansas City School of Medicine, Kansas City, MO, United States

**Keywords:** 3D-printing, commentary, meta-analysis, trauma, fractures—bone

## Introduction

Traumatic fractures are a leading cause of morbidity, mortality, and healthcare cost worldwide (1–3). The traditional management of such fractures is reduction and fixation (4), and often utilize two-dimensional (2D) imaging techniques such as digital radiography (DR), computerized tomography (CT), and magnetic resonance imaging (MRI) (5). These imaging modalities are limited in displaying the complexity of bone fractures, so there is an awareness of a legitimate risk for adverse outcomes such as prolonged intraoperative time and blood loss (5).

Moreover, three-dimensional (3D) printing is one manner to generate models of traumatic fractures to provide visual and tactile clarity to lessen adverse outcomes (6). Studies have shown that 3D models afford surgeons a superior preoperative plan, allowing them to visualize fracture morphology, choose the best approach, plan placement of screws, and communicate with the healthcare team as well as the patient, among other benefits (6–8). Recent studies have shown that 3D printing-assisted surgeries are superior to 2D imaging-assisted surgeries (8–10). Their surgical outcomes merit further investigation.

## 3D Printing-Assisted Surgeries for Traumatic Fracture Improving Patient Outcomes

The recent publication by Yang et al. (11) is a meta-analysis including 12 randomized control trials (RCTs) comparing the outcomes of 3D-printing-assisted surgery with conventional surgery for traumatic fracture.

In summary, this study utilized PubMed, Embase, and Cochrane Library for searches of RCTs that included 3D printing. Outcome data included operation duration, intraoperative blood loss, intraoperative fluoroscopy, fracture union time, and rate of excellent outcomes.

The data from 12 RCTs involving 641 patients was collected and statistically analyzed, and further subgroup analysis was done by fracture type (i.e., limb, trunk fractures).

The aggregate findings by Yang et al. had indicated that 3D printing-assisted surgeries had briefer operation duration in addition to decreased intraoperative blood loss. Moreover, a key finding by Yang et al. was supporting the notion that these treatment modalities had a higher rate of excellent outcomes compared to the with 2D-cohort. Ultimately, the meta-analysis demonstrated improved outcomes for 3D-assisted surgeries when compared to 2D-assisted surgeries for traumatic fracture.Of note, additional benefits such as improved communication with the healthcare team as well as the patient or improved placement of screws were not included in analysis.

## Discussion

The meta-analysis provided by Yang et al. is a sound design. Further data on the rates of improved communication within the healthcare team as well as with the patient would benefit further understanding of the qualitative benefits of 3D printed-assisted surgery. One consideration is that the vast majority of the included RCTs were conducted in China, which may limit the external variability of results when compared to other countries to do guidelines of treatment. However, Yang et al. does address the presence of heterogeneity within the compiled data. Another point to consider is that the meta-analysis refers to “excellent outcomes” as a variable for comparison but does not provide further elaboration as to what this entails, as some may desire.

## Notable Future Directions

One area not mentioned in the meta-analysis was the ratio of cost to benefit. The cost of 3D printing assisted-spinal surgeries has been previously described as relatively inexpensive (12). However, the impacts of 3D printing on the cost of radiologic consults for patients merits further study (13). In this manner, it would be useful to compare the cost of 3D-printing assisted surgery with traditional surgery, considering beneficial savings as well.

From the perspective of patient outcomes, there is a growing amount of literature that uses return to function as a measure of the quality of 3D printing-assisted surgeries. For example, comparisons of 3D printing for traumatic acetabular fracture utilized return to hip function as a measure (14). Yang et al. allude to this further direction by referring to “excellent outcomes” in the post-operative management. Given that patients are followed in the post-operative period, it may be possible to quantify return to function via measures such as force exerted via the joint repaired. In addition, one potential excellent outcome which may have been alluded to was decreased intraoperative blood loss compared to the traditional cohort, which is commonly used to as a parallel correlate to how invasive a procedure was (15). Moreover, from a clinical perspective, it is worth noting that the findings of this meta-analysis, while extremely sound, should ought to be used with additional considerations specific to the patient. For example, the generalization of these results may vary if the patient has a history of bone deformities, in addition to other comorbidities.

On the other hand, the authors of this commentary envision that more robust and generalizable results could be gained from such meta-analyses via the inclusion of large, multi-center global RCTs. The RCTs included by Yang et al. and conducted thus far have been single center with relatively fewer patients. Upon conduction of such studies, the methodology by Yang et al. could be used in comparing 3D printing-assisted surgeries with traditional surgeries for traumatic fracture among patients around the world.

## Conclusion

Overall, implementation of 3D printing-assisted surgeries for traumatic fractures requires a mounting amount of evidence before it is considered the standard of care. Despite this, the sound methodology provided by Yang et al. (11), Mitsouras et al. (16), and Cao et al. (17) provides a foundational level of data for the implementation of such tools, as well as engenders further study into associated costs, return to function, and involvement of international patients.

## Author Contributions

All authors listed have made a substantial, direct and intellectual contribution to the work, and approved it for publication.

## Funding

SS and KV were recipients of the Sarah Morrison Research Award at the University of Missouri—Kansas City School of Medicine.

## Conflict of Interest

The authors declare that the research was conducted in the absence of any commercial or financial relationships that could be construed as a potential conflict of interest.

## Publisher's Note

All claims expressed in this article are solely those of the authors and do not necessarily represent those of their affiliated organizations, or those of the publisher, the editors and the reviewers. Any product that may be evaluated in this article, or claim that may be made by its manufacturer, is not guaranteed or endorsed by the publisher.

## Abbreviations

2D: two-dimensional
DR: digital radiography
CT: computerized tomography
MRI: magnetic resonance imaging
3D: three-dimensional
RCTs: randomized control trials
SMD: standardized mea n differences
OR: odds ratios
CI: confidence interval.
